# Antioxidant Polyphenols of *Antirhea borbonica* Medicinal Plant and Caffeic Acid Reduce Cerebrovascular, Inflammatory and Metabolic Disorders Aggravated by High-Fat Diet-Induced Obesity in a Mouse Model of Stroke

**DOI:** 10.3390/antiox11050858

**Published:** 2022-04-27

**Authors:** Janice Taïlé, Matthieu Bringart, Cynthia Planesse, Jessica Patché, Philippe Rondeau, Bryan Veeren, Patricia Clerc, Anne Gauvin-Bialecki, Steeve Bourane, Olivier Meilhac, David Couret, Marie-Paule Gonthier

**Affiliations:** 1 Université de La Réunion, INSERM, UMR 1188 Diabète Athérothrombose Thérapies Réunion Océan Indien (DéTROI), 97490 Sainte-Clotilde, La Réunion, France; janice.taile@univ-reunion.fr (J.T.); matthieu.bringart@inserm.fr (M.B.); cynthia.planesse@univ-reunion.fr (C.P.); jessica.patche@univ-reunion.fr (J.P.); philippe.rondeau@univ-reunion.fr (P.R.); bryan.veeren@univ-reunion.fr (B.V.); steeve.bourane@inserm.fr (S.B.); olivier.meilhac@inserm.fr (O.M.); david.couret@chu-reunion.fr (D.C.); 2 UR 2212 Laboratoire de Chimie et de Biotechnologie des Produits Naturels (ChemBioPro), Université de La Réunion, CEDEX 9, 97744 Saint-Denis, La Réunion, France; patricia.clerc@univ-reunion.fr (P.C.); anne.bialecki@univ-reunion.fr (A.G.-B.); 3 CHU de La Réunion, 97410 Saint-Pierre, La Réunion, France

**Keywords:** stroke, obesity, hyperglycemia, inflammation, oxidative stress, antioxidant polyphenols

## Abstract

Metabolic disorders related to obesity and type 2 diabetes are associated with aggravated cerebrovascular damages during stroke. In particular, hyperglycemia alters redox and inflammatory status, leading to cerebral endothelial cell dysfunction, blood–brain barrier (BBB) disruption and brain homeostasis loss. Polyphenols constitute the most abundant dietary antioxidants and exert anti-inflammatory effects that may improve cerebrovascular complications in stroke. This study evaluated the effects of the characterized polyphenol-rich extract of *Antirhea borbonica* medicinal plant and its major constituent caffeic acid on a high-fat diet (HFD)-induced obesity mouse model during ischemic stroke, and murine bEnd3 cerebral endothelial cells in high glucose condition. In vivo, polyphenols administered by oral gavage for 12 weeks attenuated insulin resistance, hyperglycemia, hyperinsulinemia and dyslipidemia caused by HFD-induced obesity. Polyphenols limited brain infarct, hemorrhagic transformation and BBB disruption aggravated by obesity during stroke. Polyphenols exhibited anti-inflammatory and antioxidant properties by reducing IL-1β, IL-6, MCP-1, TNF-α and Nrf2 overproduction as well as total SOD activity elevation at the cerebral or peripheral levels in obese mice. In vitro, polyphenols decreased MMP-2 activity that correlated with MCP-1 secretion and ROS intracellular levels in hyperglycemic condition. Protective effects of polyphenols were linked to their bioavailability with evidence for circulating metabolites including caffeic acid, quercetin and hippuric acid. Altogether, these findings show that antioxidant polyphenols reduced cerebrovascular, inflammatory and metabolic disorders aggravated by obesity in a mouse model of stroke. It will be relevant to assess polyphenol-based strategies to improve the clinical consequences of stroke in the context of obesity and diabetes.

## 1. Introduction

Stroke is responsible for long-term disabilities, severe morbidity and mortality worldwide [1]. Ischemic stroke is caused by a blood flow reduction, generally resulting from important arterial occlusion. The main risk factors of ischemic stroke are hypertension, tobacco exposure, physical inactivity and metabolic disorders related to diabetes [2]. Various preclinical and clinical studies reported that obesity aggravates ischemic brain injury and subsequently exacerbates neurological impairments, oxidative stress and inflammatory response [3,4]. Obesity also constitutes a major risk factor for insulin resistance leading to type 2 diabetes. Importantly, diabetic patients are twice as likely to develop a stroke as non-diabetic subjects [5]. It has been shown that chronic hyperglycemia contributes to blood–brain barrier (BBB) integrity disruption and aggravates cerebrovascular disorders such as hemorrhagic transformation during stroke [6,7,8]. The BBB is composed of microvascular endothelial cells interconnected by tight junctions that protect the cerebral microenvironment. Indeed, claudins, occludins and zonula occludens (ZO) defined as key tight junction proteins limit BBB permeability to low molecular mass substances [9,10]. Our previous studies contributed to demonstrate the detrimental impact of hyperglycemia on BBB integrity loss in a mouse model of stroke [11,12], as well as on cerebral endothelial cell permeability and tight junction down-regulation [13]. In diabetic condition, gelatinolytic matrix metalloproteinases (MMPs) comprising MMP-2 and MMP-9 participate in tight junction protein degradation, leading to BBB disruption [14,15]. Mechanistically, oxidative stress and inflammation may play causal roles [16,17,18]. Oxidative stress characterized by an imbalance between the production of reactive oxygen species (ROS) and their insufficient degradation by the endogenous antioxidant defense system, causes redox signaling disruption and cellular damages. Various enzymes such as superoxide dismutase (SOD), catalase and glutathione peroxidase (GPx) are involved in the antioxidant defense system [19]. Their deregulation and ROS overproduction promote endothelial dysfunction by damaging DNA, proteins, cell membranes and plasma lipids. [20,21]. Otherwise, oxidative stress associated to diabetic condition activates the nuclear factor kappa B (NFκB) transcriptional factor, leading to an increased production of chemokines/cytokines including monocyte chemoattractant protein-1 (MCP-1), interleukin-1 beta (IL-1β), tumor necrosis factor-alpha (TNF-α) and interleukin-6 (IL-6). In turn, these pro-inflammatory mediators maintain redox alterations, and the crosstalk between oxidative stress and inflammation may exacerbate endothelial cell dysfunction [22,23].

Based on the evidence of the deleterious role of oxidative stress and inflammation on endothelial cell dysfunction and BBB disruption during stroke, the development of antioxidant strategies is of high interest [24,25]. Growing literature data report that plant polyphenols are able to exert strong antioxidant and anti-inflammatory effects [26,27,28]. Polyphenols represent the richest antioxidant sources of the human diet through the consumption of fruits, cereals, vegetables as well as medicinal plants [26,27,29], and may improve endothelial function [12,25,30,31]. We previously showed that the polyphenol-rich extract of *Antirhea borbonica*, a medicinal plant referenced in the French Pharmacopeia for antidiabetic properties, and its major constituent caffeic acid, attenuate oxidative stress, inflammatory response and permeability loss of cerebral endothelial cells cultured under hyperglycemic condition. Moreover, we found that these polyphenols reduce cerebral infarct volume, BBB disruption and neuroinflammation in a mouse model of stroke exposed to acute hyperglycemia [13,32]. Nevertheless, there is still lack of data regarding the effects of polyphenols during stroke in conditions of chronic hyperglycemia caused by obesity as in type 2 diabetic patients. Recently, we developed a mouse model of stroke exposed to high-fat diet (HFD)-induced obesity and impaired glucose tolerance related to insulin resistance [33]. In the present study, this animal model was used in order to evaluate, for the first time, the protective action of the characterized polyphenol-rich extract of *A. borbonica* or its predominant constituent caffeic acid against cerebrovascular complications of stroke, after administration in obese mice by oral gavage for 12 weeks. We determined the effects of polyphenols on systemic metabolic status, brain infarct, neurological deficit, hemorrhagic transformation, as well as on markers of BBB disruption, inflammation and oxidative stress. Plasma levels of polyphenols and related metabolites were measured to assess their bioavailability. In parallel, an in vitro model of murine bEnd3 cerebral endothelial cells exposed to high glucose concentration in the presence or not of polyphenols was used to evaluate the mechanistic link between MMPs activities, inflammation and redox markers.

## 2. Materials and Methods

### 2.1. Analysis of Polyphenols from A. borbonica Plant Extract, HFD and Normal Diet (ND)

*A. borbonica* plant (Rubiaceae) collected in Reunion Island (France) was botanically identified (voucher number RUN052-F) at the University of Reunion Island. The declaration of the use of the plant to the Nagoya Protocol for Access and Benefit-Sharing Clearing-House was achieved (ABSCH-IRCC-FR-252879-1). Leaves from *A. borbonica* plant were dried at 45 °C (airflow) and crushed to obtain a powder. The plant powder (2 g) was dissolved in 10 mL of an aqueous-acetonic solution (70%, *v*/*v*, Sigma-Aldrich, St-Louis, MO, USA). Then, the mixture was incubated at 4 °C for 90 min, centrifuged at 1400× *g* at 4 °C for 20 min, and the supernatant containing polyphenols collected, evaporated to eliminate the solvent, and stored at −80 °C until analysis. Concerning the extraction of polyphenols from the ND and HFD, 4 g of each diet was added to 20 mL of an aqueous-acetonic solution containing HCl (200 mM). After an incubation of the mixture at 4 °C for 90 min followed by a centrifugation at 1400× *g* at 4 °C for 20 min, the polyphenol-rich supernatants were collected and stored at −80 °C until analysis. The polyphenols extracted from *A. borbonica* plant, ND and HFD were identified by ultra-high-performance liquid chromatography coupled to electrospray ionization-tandem mass spectrometry (UPLC-ESI-MS-MS), according to the method previously reported [34]. Folin–Ciocalteu assay was conducted to determine the total polyphenol content of *A. borbonica* plant extract as we previously described [35]. Briefly, using a 96-well plate, 25 μL of *A. borbonica* plant extract was added to a mixture of 125 μL of Folin–Ciolcateu’s reagent (Sigma-Aldrich, St-Louis, MO, USA) and 100 μL of sodium carbonate (Sigma-Aldrich, St-Louis, MO, USA). After incubation at 54 °C for 5 min, and then at 4 °C for 5 min, the absorbance was measured at 760 nm (FLUOstar Optima, Bmg Labtech, Ortenberg, Germany). A calibration curve was built with a standard solution of gallic acid (Sigma-Aldrich, St-Louis, MO, USA) and the total polyphenol amount of *A. borbonica* plant extract calculated as g gallic acid equivalent (GAE)/100 g plant powder.

### 2.2. Animal Procedures and Experimental Design

All experimental procedures were approved by the local Ethics Committee for animal experimentation (APAFIS# 2019111816093342v1) and were performed in agreement with the French and European Community Guidelines for the Use of Animals in Research (86/609/EEC and 2010/63/EU). Ten-week-old C57BL/6 male mice (Janvier Labs, Le Genest-Saint-Isle, France) received a standard ND with 10% kcal% fat (Research Diets Inc., New Brunswick, USA) or HFD with 45% kcal% fat (Research Diets Inc., New Brunswick, USA) ad libitum, and were maintained under standard conditions of temperature, light and humidity. Mice were randomly allocated into 4 different groups. Group 1 received the ND with a distilled water supplementation serving as control (ND, n = 8). Group 2 received the HFD with a distilled water supplementation (HFD, n = 16). Group 3 and 4 received the HFD with either *A. borbonica* polyphenols diluted in distilled water (HFD + *A.b*, n = 16) or caffeic acid (Sigma-Aldrich, St-Louis, MO, USA) dissolved in distilled water (HFD + CA, n = 16), respectively. Mice were exposed to a dose of 35 mg/kg/day of polyphenols by oral gavage 3 days per week for 12 weeks. The selection of this pharmacological dose was based on similar literature studies using an average concentration ranging from 10–50 mg/kg [36,37,38,39], keeping in mind that polyphenol intake may reach 0.8–1 g/day in humans depending on the nutritional conditions [26]. During the experimental period, we performed the measurement of food intake and total body weight weekly, fasting glycemia every 15 days. To assess the development of insulin resistance, an oral glucose tolerance test (OGTT) was performed monthly. Mice were considered to exhibit chronic hyperglycemia when blood glucose levels ranged from 300–400 mg/dL. At the end of the experimental period, animals were anesthetized with Isoflurane (isoFlo^®^ Centravet, Maisons-Alfort, France) under the control of body temperature maintained at 34 °C, with a heating pad. Then, mice were exposed to a cerebral ischemia via a middle cerebral artery occlusion (MCAO) surgery, as we previously reported [33]. Briefly, MCAO procedure was performed during 90 min, by using a 7-0 silicon-coated monofilament (Doccol Corporation, Sharon, MA, USA) from the external carotid artery into the lumen of the internal carotid artery until resistance was felt, which reduces cerebral flow and confirms the occlusion [11,40]. A subcutaneous injection of Buprenorphine (0.05–0.1 mg/kg) (Buprecare^®^ Centravet, Maisons-Alfort, France) was administered for controlling analgesia. The monofilament was removed after 90 min under anesthesia in order to allow the reperfusion of the ischemic region for 20 h. After anesthesia, a subcutaneous injection of saline solution was performed to prevent dehydration. During the reperfusion period, mice were placed on individual cages with water, food and refinement [41]. The next day, mice were euthanized after anesthesia by intracardiac blood puncture, followed by an intracardiac perfusion of 20 mL saline solution. Blood centrifugation was achieved to collect plasma which was stored at −20 °C until analysis. Tissues including the brain, heart, liver and epididymal visceral adipose tissue were collected, weighed and immediately frozen in nitrogen before a storage at −80 °C until analysis. The brain was cut in a brain matrix mold into 1 mm coronal slices for cerebral infarct volume measurement. The operator was unaware of the animal group assignment during surgery and the experiments were blinded.

### 2.3. Measurement of Blood Glucose Levels

Glycemia was measured after an 8 h fasting with a hemoglucotest (Accu-Check, Roche, Meylan, France), by collecting blood sample at the tail every 15 days. OGTT was performed monthly and glycemia measurements conducted at 0, 15, 30, 45, 60, 90 and 120 min after oral gavage with glucose 30% (2.2 g/kg). Levels of blood glucose were expressed as mg/dL.

### 2.4. Protein Extraction and Quantification

Samples (100 mg) from cerebral infarcted hemisphere (IH) and non-infarcted hemisphere ((NIH) without cerebellum tissue, heart, liver and visceral adipose tissue were homogenized in lysis buffer (Tris-HCl (20 mM) pH 8.5, EDTA (1 mM), Triton X-100 (0.05%), pH 7.4). Then, tissues were lysed twice by TissueLyser II (Qiagen, Courtaboeuf, France) with one tungsten carbide ball per tube at 30 Hz for 1 min and centrifuged at 14,000× *g* for 10 min at 4 °C. Supernatants containing proteins were recovered for protein quantification by the bicinchoninic acid assay (BCA) [42] and stored at −80 °C until analysis. A calibration curve was built with a solution of standard bovine serum albumin (Sigma-Aldrich, St-Louis, MO, USA) in order to calculate the total protein concentrations.

### 2.5. Measurement of Pro-Inflammatory Markers

Plasma concentration of C-reactive protein (CRP) was determined by using a Mouse CRP ELISA kit (ab157712, Abcam, Paris, France). IL-1β, IL-6, TNF-α and MCP-1 levels were measured in the brain and visceral adipose tissue samples with specific Mouse ELISA kits (IL-1β (88-7013), IL-6 (88-7064), TNF-α (88-7324) and MCP-1 (88-7391), eBioscience, ThermoFisher Scientific, Dardilly, France). The absorbance was measured at 450–570 nm (FLUOstar Optima, Bmg Labtech, Ortenberg, Germany). Absolute absorbance values were normalized to the total protein concentrations measured in the samples of brain and adipose tissue by BCA assay.

### 2.6. Measurement of Metabolic Markers

Circulating levels of insulin, triglycerides and total cholesterol were evaluated by using specific ELISA or colorimetric kits (Mouse Insulin ELISA (10-1247-01), Mercodia, Paris, France; Triglycerides FS (1 5760 99 10 021) and Cholesterol FS (1 1350 99 10 021), DiaSys, Nivelles, Belgium). Adiponectin and leptin concentrations were evaluated in both plasma and visceral adipose tissue by using Mouse Adiponectin (ELM-Adiponectin-1) and Mouse Leptin (ELM-Leptin-1) ELISA kits, respectively (RayBiotech^®^, Peachtree Corners, GA, USA). The absorbance was determined at 450 nm (FLUOstar Optima, Bmg Labtech, Ortenberg, Germany), except for triglycerides and cholesterol assays at 500 nm. For the analysis of markers from the visceral adipose tissue, absolute absorbance values were normalized to the total protein contents evaluated by BCA assay.

### 2.7. Measurement of BBB Permeability Markers

Hemoglobin (Hb) concentration in the infarcted cerebral hemisphere and plasma level of Soluble Protein-100 β (S100β) were quantified by Mouse Hemoglobin (ab157715) and Mouse S100β (ab285283) ELISA kits (Abcam, Paris, France; MyBioSource Inc., San Diego, GA, USA), respectively. The absorbance was measured at 450 nm (FLUOstar Omega, Bmg Labtech, Ortenberg, Germany). Hb absolute absorbance values were normalized to the total protein contents measured in the infarcted cerebral hemisphere by BCA assay.

### 2.8. Measurement of Cerebral Infarct Volume

After 90 min of MCAO, mice were sacrificed and brains were removed. Brain slice sections were stained with 2,3,5-triphenyltetrazolium chloride 2% (TTC) at room temperature for 20 min to determine cerebral infarct volume. Mitochondrial dehydrogenases reduce TTC colorless solution in red 1,3,5-triphenylformazan, resulting in viable tissue staining. Indeed, brain tissues were differentiated according to the area coloration: the white-colored infarct area corresponds to dead cells and red-purple non-infarct area to viable cells. Brains were sectioned into 6 slices and each slice of 1 mm thickness was stained. Then, pictures of coronal brain sections and the measurement of the infarct volume following Swanson formula and Morris analysis were performed [43,44]. Infarct volume calculation was done according to the following formula:

$$Infarct\;volume\;\left(\%\;of\;the\;control\;hemisphere\right)=\frac{V_{CH}-V_{IC}}{V_{CH}}\;\times \;100$$

*V_CH_* corresponds to the volume of the control hemisphere (contralateral hemisphere) in all brain sections.

*V_IC_* corresponds to the volume of the viable area in the infarcted hemisphere (ipsilateral hemisphere) in all brain sections.

### 2.9. Neurological Deficit Score

The neurological deficit score was determined 24 h after MCAO, according to the score table described by Menzies et al. [45]. Briefly, score 0 corresponds to “no apparent deficit”, score 1 to “contralateral forelimb flexion when suspended by the tail”, score 2 to “decreased grip of the contralateral forelimb while tail pulled”, score 3 to “spontaneous movement in all directions; contralateral circling only if pulled by the tail”, score 4 to “spontaneous contralateral circling” and score 5 to “death after recovery from the anesthesia”.

### 2.10. Immunohistochemistry

Brain samples were dissected and fixed overnight with 4% paraformaldehyde-phosphate buffered saline (PBS). Then, they were rinsed in PBS before being cryoprotected in 30% sucrose-PBS for 48 h. Subsequently, they were embedded in Tissue-Tek O.C.T. compound (VWR, Briare, France) and frozen at −80 °C. Brains were cut in 14 μm thick coronal brain sections using the cryostat (Shandon cryotome FE, ThermoFisher Scientific, Dardilly, France) and dried for 10 min on a warming plate before rehydration in PBS for 10 min. Then, tissue samples were incubated with PBS-T (0.1% Triton X-100 diluted in PBS) for 10 min. To limit non-specific antigen accessibility, brain sections were blocked in humidified chamber for 1 h at room temperature with PBS-T/10% donkey serum before being incubated with the primary antibodies, namely goat anti-mouse glial fibrillary activated protein (GFAP, 1:500, ab53554, Abcam, Paris, France) and rat anti-mouse MCP-1 (1:125, SC-52701, Santa Cruz Biotechnology, Inc., Heidelberg, Germany) overnight at 4 °C. The day after, brain sections were washed three times with PBS-T for 10 min and co-incubated with the secondary antibodies of donkey anti-goat 488 (1:500, A-11055, ThermoFisher Scientific, Les Ulis, France) and donkey anti-rat 594 (1:500, A-21209, ThermoFisher Scientific, Les Ulis, France) at room temperature for 1 h, and cell nuclei were counterstained with 4′,6-diamidino-2-phenylindole (DAPI, 1:1000, D1306, ThermoFisher Scientific, Les Ulis, France). Subsequently, brain sections were washed three times for 10 min with PBS-T before being mounted with the antifading medium Vectashield (H-1000, Vector Laboratories, Burlingame, CA, USA). Brain section slides were sealed and scanned by NanoZoomer S60 Digital slide scanner C13210 (Hamamatsu Photonics, Massy, France). The calculation of the percentage of immunostaining area/total of analyzed brain area for GFAP and MCP-1 was performed blind to the treatment of the animals by using ImageJ Software with thresholds set according to signal intensity.

### 2.11. Western Blot Analysis

Proteins obtained from brain homogenates (20 μg) were separated using a sodium dodecyl sulphate-polyacrylamide gel electrophoresis with 7.5% of acrylamide and transferred onto nitrocellulose membrane. Next, a blocking of nitrocellulose membrane was performed with 5% (*w*/*v*) non-fat dry milk in Tris-buffer containing 0.1% (*v*/*v*) Tween 20 (TBS-Tween) for 1 h at room temperature before an incubation with the primary rabbit antibodies (Abcam, Paris, France) against mouse ZO-1 (1:500, ab216880), nuclear factor erythroid 2-related factor 2 (Nrf2, 1:500, ab31163) and β-actin (1:5000, ab8227) overnight at 4 °C. The day after, after washing with TBS-Tween, nitrocellulose membrane was incubated with the corresponding goat anti-rabbit secondary antibodies conjugated to horseradish peroxidase (1:2000, ab6721, Abcam, Paris, France). Chemiluminescence kit (GE Healthcare Life Sciences, Velizy-Villacoublay, France) and Amersham Imager 600 (GE Healthcare Life Sciences, Velizy-Villacoublay, France) were used for protein/antibody complex detection. Data were normalized to β-actin protein levels.

### 2.12. Measurement of the Total SOD Activity

The total SOD activity was evaluated in protein homogenates obtained from the brain, heart, liver and visceral adipose tissue by measuring acetylated cytochrome C reduction by superoxide radicals generated by the xanthine/xanthine oxidase system, with the method published by Bitar et al. [46]. The total SOD activity was assessed in a reagent buffer at pH 7.8 containing cytochrome C (0.2 mM), EDTA (2 mM), KH_2_PO_4_ (50 mM), xanthine oxidase (1–2 U/mg protein) and xanthine (0.5 mM). Every 5 s for 3 min at 25 °C, the enzymatic activities were measured at a wavelength of 550 nm (FLUOstar Omega, Bmg Labtech, Ortenberg, Germany) and calculated as U/g proteins.

### 2.13. Detection of Polyphenols and Related Metabolites in Plasma

Polyphenols were extracted from plasma according to the method previously described [47], with slight modifications. Briefly, 55 μL of acidified plasma (50 μL of plasma and 5 μL of 0.58 mM acetic acid) were spiked with 2 μL of the internal standard syringic acid (1 μM). Then, 2 μL of *Helix pomatia* extract containing sulfatase and β-glucuronidase (Sigma, St. Louis, MO, USA) were added to deconjugate metabolites. After incubation at 37 °C for 45 min, 140 μL of methanol/HCl (200 mM) were added. Samples were centrifuged (14,000× *g*, 4 min, 4 °C) and supernatants containing polyphenols were analyzed by ultra-high-performance liquid chromatography coupled with diode array detection and Heated Electrospray Ionization (HESI)-Orbitrap mass spectrometer (Q Exactive Plus, Thermo Fisher Scientific, Les Ulis, France). A volume of 10 μL of samples was injected through Thermo Fisher Ultimate 3000 series WPS-3000 RS autosampler and separated on a pentafluorophenyl column (2.6 μm, 100 mm × 2.1 mm, Phenomenex, Le Pecq, France). The elution was performed with 0.1% formic acid in water (A) and 0.1% formic acid in acetonitrile (B) at 30 °C under a flow of 0.45 mL min^−1^, according to the gradient 0–0.1min 5% B, 0.1–7.1 min 35% B, 7.1–7.9 min 95% B and 7.9–10 min 5% B. For the mass spectrometry analysis, HESI source (HESI II) was used, under nitrogen drying gas, spray voltage 2.8 kV, capillary temperature 350 °C, sheath gas flow rate 60 units, auxiliary gas flow rate 20 units and S-lens RF level 50. Registration of mass spectra was done in full scan mode (*m*/*z* 100 to 1500) under negative ion mode, with a resolving power of 7 × 10^4^ FWHM (at *m*/*z* 400). Automatic gain control was at 1 × 10^6^ and the injection time at 200 ms. Orbitrap performance was assessed weekly and external calibration performed by using LTQ ESI negative ion calibration solution (Pierce, ThermoFisher Scientific, Les Ulis, France). Polyphenols were identified according to their retention time, exact mass and MS/MS analysis. XCalibur 4.2.47 software (Thermo Fisher Scientific Inc., Les Ulis, France) and Skyline 21.1.0.146 software (MacCoss lab, Washington, DC, USA) were used for data acquisition. For quantitation, solutions containing standard quercetin, caffeic, ferulic, chlorogenic, hippuric and syringic acids (Sigma-Aldrich, St-Louis, MO, USA) were prepared in methanol and diluted in 0.1% aqueous formic acid to generate concentrations that ranged 0.005–10 μM. In each batch, two quality control samples were analyzed in duplicate, followed by two blank samples containing methanol. The intensity of each peak was plotted against the related standard concentration in order to obtain the calibration curve. Syringic acid internal standard recovery was calculated similarly. Calibration curves were built according to the method previously published [47].

### 2.14. Measurement of ROS Level, MCP-1 Secretion and Activities of Secreted MMPs in bEnd3 Cerebral Endothelial Cells in Hyperglycemic Condition

Murine bEnd3 cerebral endothelial cells (The American Type of Culture Collection, ATCC^®^ CRL-2299™, Manassas, VA, USA) were cultured in Dulbecco’s Modified Eagle Medium (DMEM) composed of 25 mM glucose, 5 mM L-glutamine, 10% heat-inactivated fetal bovine serum, 2 µg/mL streptomycin and 50 µU/mL penicillin (Pan Biotech, Dutscher, Brumath, France) in an incubator at 37 °C and 5% of CO_2_. The fluorogenic probe 2′,7′-dichlorodihydrofluorescein diacetate (DCFH-DA) assay was used to determine intracellular ROS levels, in agreement with the method previously described [32]. Briefly, bEnd3 cells were cultured in a 96-well black plate (1.8 × 10^4^ cells/well) in DMEM that contained 5.5 mM glucose (normoglycemic condition, NG) for 24 h. After medium removal, bEnd3 cells were rinsed twice with PBS and exposed to 100 μL per well of 10 μM of DCFH-DA diluted in PBS (Sigma-Aldrich, St-Louis, MO, USA) in a humidified atmosphere (5% CO_2_, 37 °C) for 45 min. After the removal of PBS containing DCFH-DA, cells were exposed either to polyphenol-rich extract of *A. borbonica* plant (10 µM GAE) or pure quercetin, caffeic acid or chlorogenic acid (10 µM) in hyperglycemic condition (HG, 33 mM glucose) or in NG for 3 h. Noteworthy, the dose of 10 µM of polyphenols was selected in line with our published studies on cerebral endothelial cells exposed to polyphenols at this pharmacological dose, as broadly used in similar experiments reported in the literature [12,13,32]. Even if in vivo polyphenol bioavailability may depend on the interindividual variability and may differ from one polyphenol to another, circulating polyphenol concentrations reach the range of μM in nutritional interventions [26]. To quantify intracellular ROS levels, a measurement of probe fluorescence was performed at the excitation and emission wavelengths of 492 nm and 520 nm, respectively (FLUOstar Optima, Bmg Labtech, Cambridge, UK). Concerning MCP-1 secretion analysis, bEnd3 cells were cultured in a 6-well plate (3.5 × 10^5^ cells/well) in NG for 24 h. Then, the removal of medium was realized and bEnd3 cells exposed for 24 h to polyphenol-rich extract of *A. borbonica* plant (10 µM GAE) or pure quercetin, caffeic acid or chlorogenic acid (10 µM) in HG or NG conditions. Then, cell culture media were sampled and analyzed using Mouse MCP-1 ELISA kit (eBioscience, ThermoFisher Scientific, Dardilly, France). To extract the cellular proteins, a volume of 700 μL of PBS was added to each well. After scrapping, bEnd3 cells were collected and centrifuged at 900× *g* for 4 min at 25 °C. Supernatants were discarded and a volume of 200 μL of lysis buffer (25 mM Tris pH 8.3, 10 mM KCl, 1 mM DTT, 1 mM EDTA, 1% Triton X-100, protease inhibitors 1 X) added. After resuspension of the cellular pellet in the lysis buffer, a centrifugation was done at 900× *g* for 4 min at 25 °C, and supernatants containing proteins collected. Protein concentrations were measured with BCA assay at 450–570 nm (FLUOstar Optima, Bmg Labtech, Ortenberg, Germany). A normalization of absolute absorbance to protein quantity was performed. For the measurement of secreted MMP-2 and MMP-9 activities, zymography was performed on conditioned media and cell lysates as previously described [48,49]. Briefly, bEnd3 cells were cultured in a 6-well plate at a density of 3.5 × 10^5^ cells per well in NG during 24 h. Then, the removal of the medium was realized and bEnd3 cells exposed for 24 h to polyphenol-rich extract of *A. borbonica* plant (10 µM GAE) or pure quercetin, caffeic acid or chlorogenic acid (10 µM) in HG or NG conditions. Next, for each condition, an equal volume of conditioned media was loaded onto a 7.5% polyacrylamide gel. After electrophoresis, gels were washed twice in a 2.5% Triton X-100 solution for 30 min and rinsed with several distilled water baths. The gels were then incubated for 19 h in a buffer containing Tris (50 mM) and CaCl2 (2.5 mM) with gentle agitation at room temperature before staining with Coomassie Blue for at least 4 h. Next, the zymography gels were destained in a solution of 10% of acetic acid and 30% of ethanol, in order to visualize and quantify the bands that corresponded to the active forms of MMP-2 and 9. The calculation of MMP-2 and 9 activities was performed as previously described [48,49].

### 2.15. Statistical Analysis

Data were expressed as means ± SEM. Statistical analysis under GraphPad Prism 6 program (GraphPad Software, Inc., San Diego, CA, USA), was conducted to control data normality and homogeneity, and then based on an analysis of variance (ANOVA) associated with Bonferroni’s multiple comparison test. Pearson linear correlation was built when appropriate. Differences were statistically significant for a *p* value < 0.05.

## 3. Results

### 3.1. Identification of Polyphenols Extracted from A. borbonica Plant, ND and HFD

**Figure 1:**
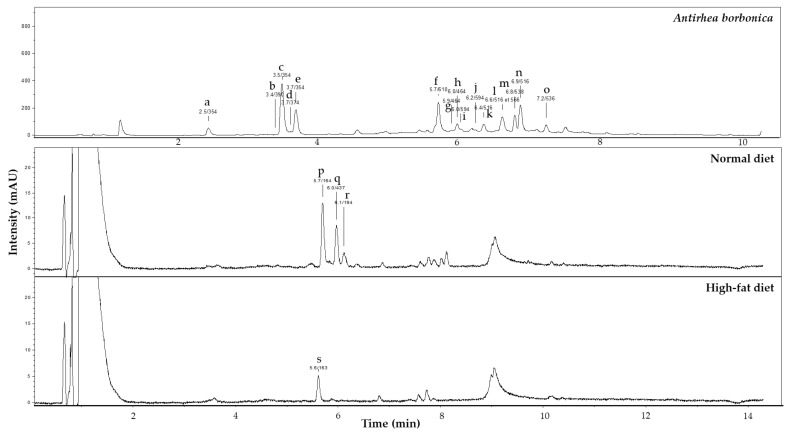
Polyphenolic composition of *A. borbonica* plant extract, normal diet and high-fat diet. Mass spectrometry analysis was performed to identify and quantify polyphenols present in *A. borbonica* medicinal plant extract, normal and high-fat diets at 320 nm. The detection of compounds was based on their retention time and the *m*/*z* ratio of their parent ions and fragments in negative mode. In *A. borbonica* plant extract, the compounds detected were (**a**,**c**,**e**): chlorogenic acid isomers, (**b**): monotropein, (**d**): unidentified molecule, (**f**): quercetin-hexose-rhamnose; (**g**,**h**): quercetin-hexose; (**i**,**j**): kaempferol-hexose-rhamnose; (**k**,**l**,**n**): dicaffeoylquinic acid isomers; (**m**): coumaroyl-dihydromonotropein; (**o**): coumaroyl-monotropein. In the normal diet, the compounds found were (**p**): coumaric acid, (**q**): unidentified molecule; (**r**): ferulic acid. In the high-fat diet, the compound detected was (**s**): coumaric acid.

UPLC-MS-MS analysis was performed to identify polyphenols present in *A. borbonica* plant extract, ND and HFD. Data reported in Figure 1 indicate that the most abundant polyphenols identified in *A. borbonica* plant extract were isomers of chlorogenic acid (2292.5 ± 29.4 µg/mL) and dicaffeoylquinic acid (1065.3 ± 15.8 µg/mL) which are caffeic acid esters belonging to the family of dietary phenolic acids. Other phenolic acids deriving from coumaric acid were depicted at a lower concentration (152.3 ± 2.6 µg/mL). Flavonoids including glycosylated derivatives of quercetin (753.1 ± 10.6 µg/mL) and kaempferol (40.5 ± 1.6 µg/mL) were also detected in *A. borbonica* plant extract. In parallel, few quantities of coumaric acid (0.51 ± 0.01 µg/mL) and ferulic acid corresponding to the methylated form of caffeic acid (0.17 ± 0.006 µg/mL) were identified in ND, while only coumaric acid could be quantified in HFD (0.19 ± 0.005 µg/mL). These results demonstrate that the phenolic acids such as caffeic acid esters and the flavonoids such as quercetin and kaempferol were polyphenols specifically provided by *A. borbonica* plant extract, as compared to coumaric and ferulic acids present in ND and HFD, and known to originate from the wheat flour composing these diets. Of note, given that derivatives of caffeic acid were depicted as the major phenolic acids present in *A. borbonica* extract, and based on our previous study showing the detection of caffeic acid in the brain of a mouse model of stroke exposed to *A. borbonica* extract [12], standard caffeic acid was used in comparison to *A. borbonica* polyphenol-rich extract in the following experiments.

### 3.2. Effect of HFD and Polyphenols on Food Intake, Body and Tissue Weights, and Fat Deposits

**Figure 2:**
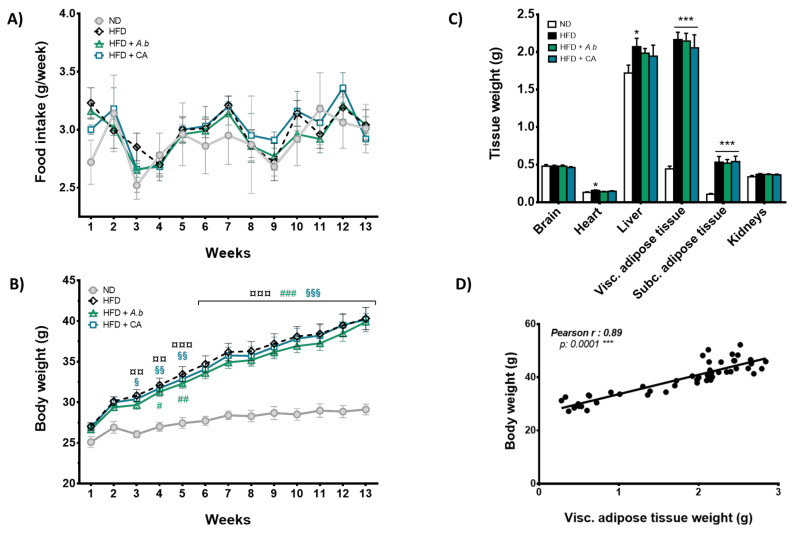
Effect of HFD and polyphenols on food intake, body and tissue weights, and fat deposits. Mice were exposed to a normal diet (ND) or a high-fat diet (HFD) in the presence or not of *A. borbonica* polyphenolic extract (HFD + *A.b*; 35 mg per kg body weight) or caffeic acid (HFD + CA; 35 mg per kg body weight) for 12 weeks before cerebral ischemia-reperfusion: (**A**) Food intake and (**B**) body weight were measured every week through 12 weeks; (**C**) weights of the brain, heart, liver, kidneys and both visceral (visc) and subcutaneous (subc) adipose tissues were measured at the end of the experimental period; (**D**) statistical correlation between total body weight and visceral adipose tissue weight was determined. Data were expressed as means ± SEM of n ≥ 5. ^¤¤^ *p* < 0.01 and ^¤¤¤^ *p* < 0.005 for HFD vs. ND. ^#^ *p* < 0.05, ^##^ *p* < 0.01 and ^###^ *p* < 0.005 for HFD + *A.b* vs. ND. ^§^ *p* < 0.05, ^§§^ *p* < 0.01 and ^§§§^ *p* < 0.005 for HFD + CA vs. ND. * *p* < 0.05 and *** *p* < 0.005 vs. ND.

The impact of HFD combined or not with polyphenols on the food intake was examined weekly for 12 weeks. Data show that the average daily food consumption of mice was not significantly modified by the diets (Figure 2A). However, mice exposed to HFD exhibited a higher total body weight than animals receiving ND (Figure 2B). Neither polyphenol-rich extract from *A. borbonica* plant nor caffeic acid altered the weight gain induced by HFD. Given that the food intake was unchanged, these results demonstrate that the increase in the total body weight of mice was related to the consumption of HFD. Consistently, HFD induced a significant elevation of the total weight of organs including the adipose tissues, liver and heart that were collected at the end of the 12-week experimental period (Figure 2C). Noticeably, HFD led to a 5-fold increase in both visceral and subcutaneous fat deposits, and a strong positive correlation between the total body weight and visceral fat deposits was depicted (Figure 2D). Altogether, these results suggest that HFD promoted obesity through an accumulation of fat deposits, and polyphenols did not modify HFD-induced fat gain.

### 3.3. Effect of HFD and Polyphenols on Glycemia, Insulinemia and Lipidemia

**Figure 3:**
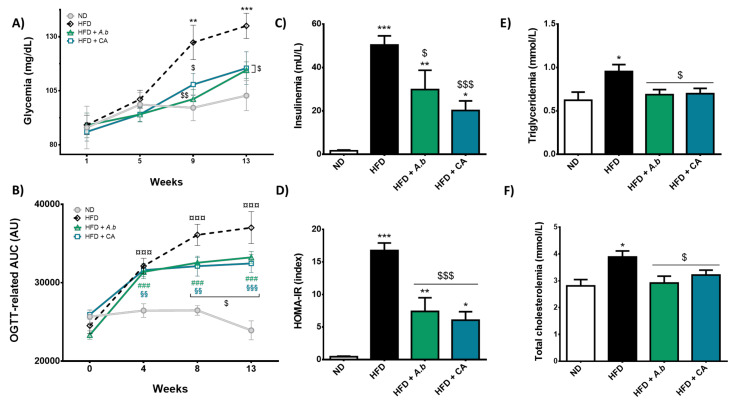
Effect of HFD and polyphenols on glycemia, insulinemia and lipidemia. Mice were exposed to a normal diet (ND) or a high-fat diet (HFD) in the presence or not of *A. borbonica* polyphenolic extract (HFD + *A.b*; 35 mg per kg body weight) or caffeic acid (HFD + CA; 35 mg per kg body weight) for 12 weeks before a cerebral ischemia-reperfusion: (**A**) Glycemia and (**B**) area under curve index (AUC) were measured. After a cerebral ischemia of 90 min and 20 h of reperfusion, (**C**) insulinemia was evaluated by ELISA kit and (**D**) HOMA-IR was calculated; (**E**) Circulating levels of triglycerides and (**F**) total cholesterol were determined by colorimetric assays. Data were expressed as means ± SEM of n ≥ 5. * *p* < 0.05, ** *p* < 0.01 and *** *p* < 0.005 vs. ND. ^$^ *p* < 0.05, ^$$^ *p* < 0.01 and ^$$$^ *p* < 0.005 vs. HFD. ^¤¤¤^ *p* < 0.005 for HFD vs. ND. ^###^ *p* < 0.005 for HFD + *A.b* vs. ND. ^§§^ *p* < 0.01 and ^§§§^ *p* < 0.005 for HFD + CA vs. ND.

We assessed whether HFD-induced obesity was associated with systemic metabolic disorders. Results show that fasting blood glucose levels measured in HFD-exposed mice were significantly higher than those determined in the control ND group (Figure 3A), after a 9-week experimental period and until the end of the experiment. Interestingly, exposure of mice to A. borbonica polyphenols or caffeic acid significantly reduced glycemia from 135.0 mg/dL in the HFD group to 114.5 and 115.4 mg/dL at the end of the experimental period, respectively. Concordantly, polyphenols significantly attenuated HFD-associated glucose intolerance measured monthly by OGTT assay (Figure 3B). Moreover, while HFD led to an up-regulation of insulinemia (Figure 3C) and HOMA-IR index (Figure 3D), A. borbonica polyphenols and caffeic acid significantly decreased these markers of insulin resistance. These results suggest the capacity of polyphenols to limit hyperglycemia, hyperinsulinemia and insulin resistance in obese mice. Additionally, polyphenols improved dyslipidemia, by reducing the circulating levels of triglycerides (Figure 3E) and total cholesterol (Figure 3F) that were increased by HFD.

### 3.4. Effect of HFD and Polyphenols on Leptinemia, Adiponectinemia and Plasma CRP Levels

**Figure 4:**
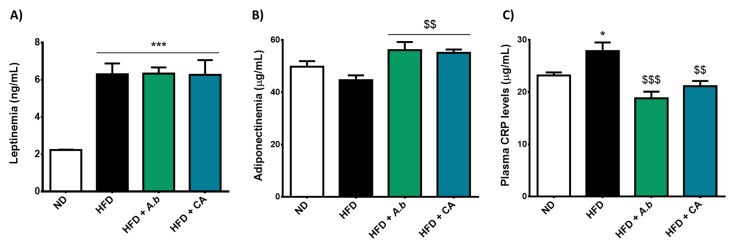
Effect of HFD and polyphenols on leptinemia, adiponectinemia and plasma CRP levels. Mice were exposed to a normal diet (ND) or a high-fat diet (HFD) in the presence or not of *A. borbonica* polyphenolic extract (HFD + *A.b*; 35 mg per kg body weight) or caffeic acid (HFD + CA; 35 mg per kg body weight) for 12 weeks before a cerebral ischemia-reperfusion. After a cerebral ischemia of 90 min and 20 h of reperfusion, (**A**) leptinemia, (**B**) adiponectinemia and (**C**) plasma CRP levels were determined by ELISA kits. Data were expressed as means ± SEM of *n* ≥ 5. * *p* < 0.05 and *** *p* < 0.005 vs. ND. ^$$^ *p* < 0.01 and ^$$$^ *p* < 0.005 vs. HFD.

Both leptin and adiponectin constitute major adipokines produced by the adipose tissue and are inversely regulated in obesity. While leptinemia is increased during the accumulation of fat deposits, there is a negative link between adiponectinemia and fat gain [50]. Moreover, if leptin plays a pro-inflammatory role during obesity, adiponectin exerts an anti-inflammatory action, and its decrease in obese patients exacerbates obesity-induced inflammation. CRP is a pro-inflammatory marker secreted by the liver whose levels have been reported to be elevated during obesity [51]. We determined the impact of HFD-induced obesity, combined or not with polyphenols on leptinemia (Figure 4A), adiponectinemia (Figure 4B) and plasma CRP levels (Figure 4C) in mice. Data show that obese mice exhibited higher circulating levels of leptin and CRP than those of control mice. Moreover, HFD-induced obesity tended to decrease adiponectinemia without reaching statistical significance. Polyphenols did not modulate hyperleptinemia. Importantly, they abrogated the elevation of CRP plasma levels and up-regulated adiponectinemia in obese mice. Thus, these results suggest a link between the exposure to polyphenols and the improvement of the systemic anti-inflammatory profile in obese mice, through lowered CRP levels and raised adiponectinemia.

### 3.5. Effect of HFD and Polyphenols on Brain Infarct Volume and Neurological Deficit Score

**Figure 5:**
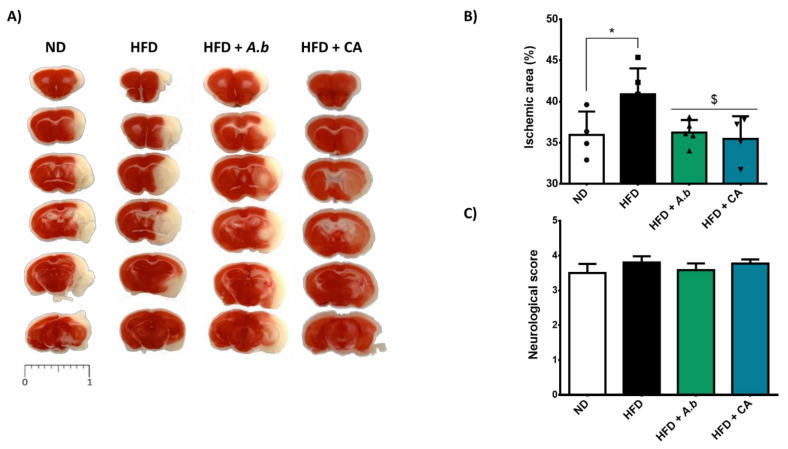
Effect of HFD and polyphenols on brain infarct volume and neurological deficit score. Mice were exposed to a normal diet (ND) or a high-fat diet (HFD) in the presence or not of *A. borbonica* polyphenolic extract (HFD + *A.b*; 35 mg per kg body weight) or caffeic acid (HFD + CA; 35 mg per kg body weight) for 12 weeks before a cerebral ischemia-reperfusion. After a cerebral ischemia of 90 min and 20 h of reperfusion, (**A**) the cerebral infarct volume (white area) was measured on brain sections after TTC vital staining and (**B**) quantified as a percentage of the cerebral infarcted hemisphere; (**C**) The neurological deficit was determined and expressed as a score ranging from 0 to 5. Data were expressed as means ± SEM of *n* ≥ 5. * *p* < 0.05 vs. ND. ^$^ *p* < 0.05 vs. HFD.

To determine the effect of HFD combined or not with polyphenols on brain infarct volume of obese mice exposed to stroke, TTC vital staining was performed. The cerebral infarct volume was measured (Figure 5A, white zone) and expressed as a percentage of the infarcted hemisphere (Figure 5B). Data indicated a higher brain infarct volume reaching 40.9% in obese mice vs. 36.0% in control animals. Importantly, the supplementation with *A. borbonica* polyphenols or caffeic acid counteracted the increase in cerebral infarct volume observed in obese mice. Results show that cerebral ischemia-reperfusion performed to mimic stroke induced a severe neurological deficit reaching an average score of 3.5 in mice (Figure 5C). However, the use of this global score did not reveal statistically significant differences in animals fed ND and HFD combined or not with polyphenols. Likewise, there was not statistical difference between the experimental groups for the mortality rate reaching 8.9% (5 mice among the 56 animals exposed to stroke). Altogether, these results suggest that polyphenols attenuated the aggravation of brain infarct in a mouse model of stroke under obesity conditions, without modulating the neurological deficit score.

### 3.6. Effect of HFD and Polyphenols on Hemorrhagic Transformation and BBB Disruption

**Figure 6:**
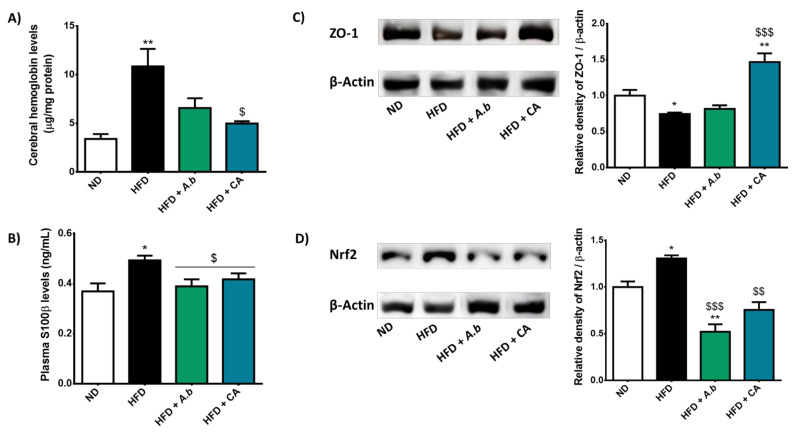
Effect of HFD and polyphenols on markers related to hemorrhagic transformation, BBB disruption and oxidative stress. Mice were exposed to a normal diet (ND) or a high-fat diet (HFD) in the presence or not of *A. borbonica* polyphenolic extract (HFD + *A.b*; 35 mg per kg body weight) or caffeic acid (HFD + CA; 35 mg per kg body weight) for 12 weeks before a cerebral ischemia-reperfusion. After a cerebral ischemia of 90 min and 20 h of reperfusion, (**A**) cerebral hemoglobin levels and (**B**) plasma S100β levels were determined by ELISA kits; (**C**) ZO-1 tight junction and (**D**) Nrf2 redox-sensitive transcriptional factor production was determined by Western blotting and normalized against β-actin housekeeping protein level in the cerebral infarcted hemisphere. Data were expressed as means ± SEM of *n* ≥ 5. * *p* < 0.05 and ** *p* < 0.01 vs. ND. ^$^ *p* < 0.05, ^$$^ *p* < 0.01 and ^$$$^ *p* < 0.005 vs. HFD.

Cerebral hemorrhagic transformation was assessed by hemoglobin quantification in the infarcted hemisphere of mice exposed to ND and HFD combined or not with polyphenols. Data show that cerebral hemoglobin levels were 3-fold higher in HFD-exposed mice than in control ND animals, suggesting that hemorrhagic transformation was exacerbated in obese mice during stroke (Figure 6A). Interestingly, while *A. borbonica* polyphenol-rich extract tended to limit the elevation of cerebral hemoglobin level in the HFD group, caffeic acid supplementation allowed reaching statistical significance. We also measured plasma concentration of S100β established as a glial-specific protein secreted by astrocytes, and used as a peripheral biomarker of BBB permeability and brain injury. Results show a higher circulating level of S100β in obese mice subjected to stroke than that measured in animals of the control ND group (Figure 6B). Exposure to *A. borbonica* polyphenols or caffeic acid significantly decreased S100β blood level in the HFD group. Moreover, caffeic acid led to an up-regulation of ZO-1 tight junction protein level that was reduced in obese mice during stroke, in contrast to *A. borbonica* polyphenolic extract without statistically significant effect (Figure 6C). These findings suggest the ability of polyphenols, and more particularly caffeic acid, to improve hemorrhagic transformation and BBB disruption markers aggravated by obesity in a mouse model of stroke.

### 3.7. Effect of HFD and Polyphenols on Brain and Visceral Adipose Tissue Inflammation

**Figure 7:**
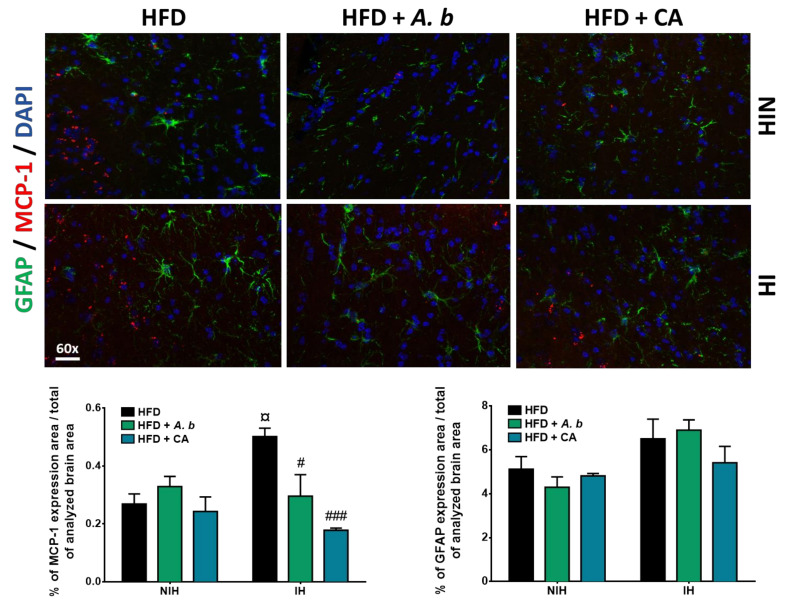
Effect of HFD and polyphenols on GFAP astrocyte marker and MCP-1 pro-inflammatory chemokine production in the brain. Mice were exposed to a normal diet (ND) or a high-fat diet (HFD) in the presence or not of *A. borbonica* polyphenolic extract (HFD + *A.b*; 35 mg per kg body weight) or caffeic acid (HFD + CA; 35 mg per kg body weight) for 12 weeks before a cerebral ischemia-reperfusion. After a cerebral ischemia of 90 min and 20 h of reperfusion, GFAP and MCP-1 were detected by immunohistochemistry in the cerebral non-infarcted hemisphere (NIH) and infarcted hemisphere (IH). Nuclei were counterstained by using DAPI. The percentage of immunostaining area/total of analyzed brain area was calculated for GFAP and MCP-1. Data were expressed as means ± SEM of *n* ≥ 3. ^¤^ *p* < 0.05 vs. HFD-NIH. ^#^ *p* < 0.05 and ^###^ *p* < 0.005 vs. HFD-IH.

Stroke is known to mediate pro-inflammatory alterations including astrocyte activation. Astrocytes are the major cerebral glial cells and exert a pivotal role in BBB function. In neuro-inflammatory conditions such as stroke, astrocyte hypertrophy has been associated with GFAP intermediary filament production used as a marker of reactive astrocytosis [52,53]. We evaluated the effect of polyphenols on GFAP protein levels in obese mice exposed to stroke. Immunohistochemical data show that GFAP staining tended to be more pronounced in the infarcted hemisphere as compared to the non-infarcted hemisphere of obese mice (Figure 7), suggesting the possibility of changes in astrocyte morphology/functionality. Supplementation with polyphenols did not significantly modulate GFAP staining rate.

To evaluate the anti-inflammatory effect of polyphenols, cerebral levels of cytokines/chemokines were quantified by ELISA and by immunohistochemical staining for MCP-1. As reported on Table 1, IL-1β, IL-6, TNF-α and MCP-1 levels were higher in the brain of mice exposed to HFD-induced obesity as compared to control animals from the ND group. The infarcted hemisphere represented the major source of neuroinflammation, and MCP-1 was detected as the most abundant pro-inflammatory marker, displaying a 5-fold higher level in obese mice. Supplementation with polyphenols limited the levels of pro-inflammatory markers that were elevated in brains of obese mice. Concordantly, in the infarcted hemisphere of obese mice, the elevation of MCP-1 staining was reduced in presence of polyphenols (Figure 7), confirming their possible protective effects on brain inflammation caused by the experimental stroke in obese mice. Noteworthy, the anti-inflammatory activity extent of polyphenols depended on the cerebral pro-inflammatory marker and the polyphenol considered. Similarly, at the visceral adipose tissue level, HFD-induced obesity led to an up-regulation of the production of IL-1β, IL-6, TNF-α and MCP-1 as well as leptin and adiponectin (Table 1). Administration of polyphenol-rich extract from *A. borbonica* plant reduced the levels of IL-6 and leptin pro-inflammatory factors while it increased that of adiponectin anti-inflammatory marker. In parallel, caffeic acid supplementation lowered the concentrations of TNF-α, IL-6 and leptin whereas it raised adiponectin levels. This is consistent with data described above reporting an elevated adiponectinemia in mice exposed to polyphenols. Altogether, these results suggest the capacity of polyphenols to exert anti-inflammatory properties, by attenuating the cerebral and peripheral pro-inflammatory alterations aggravated by HFD-induced obesity in mice during stroke.

### 3.8. Effect of HFD and Polyphenols on Cerebral and Peripheral Oxidative Stress Markers

**Figure 8:**
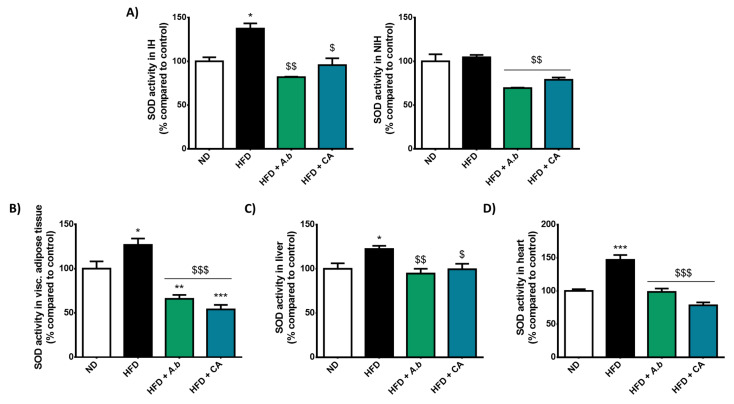
Effect of HFD and polyphenols on the cerebral and peripheral antioxidant SOD activities. Mice were exposed to a normal diet (ND) or a high-fat diet (HFD) in the presence or not of *A. borbonica* polyphenolic extract (HFD + *A.b*; 35 mg per kg body weight) or caffeic acid (HFD + CA; 35 mg per kg body weight) for 12 weeks before a cerebral ischemia-reperfusion. After a cerebral ischemia of 90 min and 20 h of reperfusion, the total SOD activity was measured (**A**) in the cerebral non-infarcted hemisphere (NIH) and infarcted hemisphere (IH), (**B**) visceral (visc) adipose tissue, (**C**) liver and (**D**) heart. Data were expressed as means ± SEM of *n* ≥ 5. * *p* < 0.05, ** *p* < 0.01 and *** *p* < 0.005 vs. ND. ^$^ *p* < 0.05, ^$$^ *p* < 0.01 and ^$$$^ *p* < 0.005 vs. HFD.

Literature data have reported a critical regulatory loop between inflammation and oxidative stress [22,23]. We assessed the impact of HFD-induced obesity and polyphenols on redox markers during stroke by evaluating the level of the protein Nrf2 known as a key redox-sensitive transcriptional factor regulating the production of redox enzymes, and the activity of SOD recognized as a first line of the endogenous enzymatic antioxidant defense system. Data show that HFD-induced obesity significantly increased the level of Nrf2 in the cerebral infarcted hemisphere (Figure 6D). Exposure to *A. borbonica* polyphenols and caffeic acid protected against Nrf2 overproduction in obese mice. Moreover, we found that the total SOD activity was significantly elevated in the cerebral infarcted hemisphere of obese mice while it was not changed in the cerebral non-infarcted hemisphere (Figure 8A). *A. borbonica* polyphenols and caffeic acid inhibited this elevation of SOD activity that was specifically mediated by obesity in the infarcted hemisphere of mice during stroke. Polyphenols also reduced the basal SOD activity measured in the non-infarcted hemisphere. Meanwhile, *A. borbonica* polyphenols and caffeic acid abrogated the increase in the total SOD activity detected in the visceral adipose tissue (Figure 8B), liver (Figure 8C) and heart (Figure 8D) of obese mice during stroke. These results demonstrate that polyphenols exhibited antioxidant properties by preserving the activation of the endogenous antioxidant system involving Nrf2 and SOD. They reduced oxidative stress aggravated by HFD-induced obesity at both the cerebral and peripheral levels during stroke.

### 3.9. Effect of Polyphenols on MMP Activities of Cerebral Endothelial Cells Exposed to a Hyperglycemic Condition

**Figure 9:**
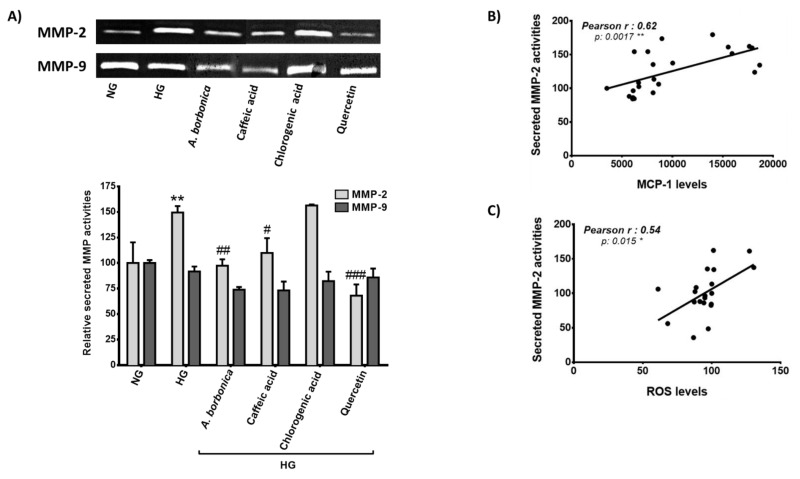
Effect of hyperglycemic condition and polyphenols on MMP activities of cerebral endothelial cells. Murine bEnd3 cerebral endothelial cells were exposed to normoglycemic condition (NG) or hyperglycemic condition (HG) in the presence or not of *A. borbonica* polyphenolic extract (10 μM GAE) or a pure polyphenol (caffeic acid, chlorogenic acid or quercetin, 10 μM). Conditioned media were used to determine (**A**) secreted MMP-2 and MMP-9 activities by zymography; (**B**) secreted levels of MCP-1 were measured by ELISA and correlated to MMP-2 activity; (**C**) intracellular levels of ROS were quantified by DCFH-DA assay and correlated to MMP-2 activity. Data are means ± SEM of *n* = 3 (3 independent cellular passages). * *p* < 0.05, ** *p* < 0.01 vs. NG. ^#^ *p* < 0.05, ^##^ *p* < 0.01 and ^###^ *p* < 0.005 vs. HG.

Our data described above show that HFD-induced obesity led to insulin resistance-mediated hyperglycemia, aggravated BBB disruption markers, as well as cerebral pro-inflammatory and redox alterations during stroke. In order to establish a mechanistic link between loss of BBB integrity, neuroinflammation and oxidative stress, we assessed the impact of hyperglycemic condition on the activity of both MMP-2 and MMP-9 known to be involved in BBB breakdown. For this experiment, murine bEnd3 cerebral endothelial cells were exposed to normoglycemic or hyperglycemic conditions in the presence or not of *A. borbonica* polyphenolic extract or pure quercetin, caffeic or chlorogenic acids detected as major polyphenols in the plant extract. Activities of secreted MMP-2 and MMP-9 were measured by zymography analysis. We found that hyperglycemic condition led to a significant increase in MMP-2 activity without changing that of MMP-9 (Figure 9A). Interestingly, the polyphenolic extract of *A. borbonica* protected against hyperglycemic condition-mediated MMP-2 activation. Quercetin and caffeic acid were able to exert a similar beneficial action, leading to suggest their possible involvement in the bioactivity of *A. borbonica* extract. Moreover, we found a significant positive correlation, on the one hand between MMP-2 activity and MCP-1 secreted level (Figure 9B), and on the other hand between MMP-2 activity and ROS intracellular level (Figure 9C). Taken together, these results raise the possibility of MMP-2 involvement in cerebral endothelial cell permeability and BBB disruption during neuroinflammation and oxidative stress aggravated by HFD in obese and hyperglycemic mice during stroke, and show the protective action of polyphenols able to target MMP-2 activity.

### 3.10. Plasma Concentrations of Polyphenols and Related Metabolites

In order to link the protective effects of polyphenols to their bioavailability, circulating concentrations of polyphenols and related metabolites were measured in mice. UPLC-MS analysis led to quantify caffeic acid and quercetin identified as two polyphenols provided by *A. borbonica* plant extract whereas chlorogenic acid present in the plant extract was not detected in mice (Table 2). Moreover, we detected ferulic acid established as one major circulating methylated metabolite of caffeic acid, as well as hippuric acid known as a predominant metabolite originating from the catabolism of polyphenols by the gut bacterial microbiota. Data show that ferulic acid levels were not modulated in mice that received *A. borbonica* polyphenols and caffeic acid. Its basal detection in mice fed an ND may be related to its presence in ND, as described above and based on the wheat flour composition of this diet. HFD-treated mice also exhibited a basal level of ferulic acid which could originate from the metabolic transformation of coumaric acid present in the HFD and also provided by the wheat flour composing this diet. Interestingly, caffeic acid concentrations were significantly elevated in mice treated with *A. borbonica* polyphenols or caffeic acid, demonstrating its capacity to be absorbed. In parallel, quercetin was specifically detected in mice treated with *A. borbonica* polyphenolic extract, showing its bioavailability. Hippuric acid levels were also significantly raised by a 10-fold factor in animals receiving the polyphenols, providing evidence for its production from the gut microbiota catabolism of *A. borbonica* polyphenols and caffeic acid.

## 4. Discussion

This study demonstrates that the polyphenol-rich extract of the medicinal plant *A. borbonica* and its major constituent caffeic acid exerted protective metabolic, anti-inflammatory and cerebrovascular effects in a mouse model of stroke under HFD-induced obesity condition. First, data show that the exposure to HFD for 12 weeks led to a 2-fold increase in the total body weight of mice, without impact of polyphenols on fat gain and food consumption. The elevation of adiposity caused by HFD was associated with an accumulation of fat mass in both the visceral and subcutaneous adipose tissues, liver and heart. In accordance with published data, our results show that obese mice exhibited an increase in glycemia, insulinemia, HOMA-IR index indicating insulin resistance, triglyceridemia, cholesterolemia, leptinemia and plasma CRP levels [33]. Interestingly, administration of polyphenols from *A. borbonica* or caffeic acid reduced these systemic metabolic and inflammatory disorders, except for leptinemia, and increased adiponectinemia. On the one hand, literature data describe leptin as a pro-inflammatory mediator and a risk biomarker for first hemorrhagic stroke [54,55]. On the other hand, adiponectin is an antioxidant, anti-inflammatory and insulin-sensitizing adipokine [56] that acts as a protective mediator during cerebral ischemia. Adiponectin^−/−^ mice were reported to exhibit an increased cerebral infarct after ischemia-reperfusion. In these mice, an addition of exogenous adiponectin limited the infarcted volume [57]. In the present study, polyphenols reduced leptin level and increased adiponectin quantity in the visceral adipose tissue of obese mice. Concordantly, polyphenols led to an elevated adiponectinemia in obese mice. The transcriptional factor peroxisome proliferator-activated receptor gamma (PPARγ) is a strong inducer of adiponectin gene expression in adipocytes [58]. We previously reported the capacity of *A. borbonica* polyphenols and caffeic acid to induce PPARγ and adiponectin production in murine 3T3-L1 adipocytes [34]. Such a positive regulatory role of polyphenols on PPARγ activation at the adipocyte level may account for the elevation of adiponectin level in the visceral adipose tissue of obese mice receiving *A. borbonica* polyphenols or caffeic acid. Taken together, these results demonstrate the protective peripheral action of polyphenols, leading to a decrease in the aggravation of metabolic and pro-inflammatory disorders in obese mice.

Our data show that polyphenols from *A. borbonica* and caffeic acid also exerted beneficial central effects during stroke. Indeed, polyphenols reduced brain infarct volume, hemorrhagic transformation (decreased cerebral level of hemoglobin), as well as plasma concentration of the glial marker S100β. Both hemoglobin and S100β are used as biomarkers of BBB breakdown. These findings suggest the capacity of polyphenols to attenuate the loss of BBB integrity aggravated in obese mice. Literature data have shown that metabolic disorders such as hyperlipidemia and hyperglycemia in obese mice contribute to BBB permeability and brain oedema formation in the acute phase of cerebral ischemia. The molecular mechanisms associated may involve lipid peroxidation and MMP activation that leads to tight junction protein degradation [3,59]. Our study demonstrates that polyphenols and more particularly, caffeic acid, protected against the decrease in ZO-1 tight junction protein level in the infarcted hemisphere of obese and hyperglycemic mice exposed to stroke. The absence of protective action of *A. borbonica* polyphenols against ZO-1 reduction may be related to the lower bioavailability of *A. borbonica* polyphenols that we measured at the blood level. Indeed, quercetin and caffeic acid circulating levels reached 0.04 and 0.9 µM, respectively, in mice fed *A. borbonica* polyphenols. In parallel, the circulating concentration of caffeic acid reached 1.2 µM in mice fed pure caffeic acid. Noteworthy, our published data reported the ability of polyphenols from *A. borbonica* and caffeic acid to counteract hyperglycemia-mediated decrease in VE-cadherin protein level in the infarcted hemisphere in a mouse model of stroke exposed to polyphenols via intraperitoneal administration [12]. Both tight junctions and adherens junction proteins such as VE-cadherin regulate BBB integrity and may be impaired during hyperglycemic condition related to diabetes [10,13]. Moreover, on an in vitro model of cerebral endothelial cells, we found that hyperglycemic condition increased the cellular permeability to FITC-Dextran marker and reduced the expression of genes encoding tight junction proteins such as ZO-1, ZO-2, occludin and claudin-5 [10,13]. Exposure of cerebral endothelial cells to *A. borbonica* polyphenols or caffeic acid attenuated the deleterious effect of hyperglycemic condition on these BBB integrity markers [13].

During BBB disruption related to stroke, an elevated production of adhesion molecules in the ischemic brain tissue of obese animal models was reported. These adhesion proteins promote the massive infiltration of inflammatory cells such as neutrophils into the brain [3,60]. Neutrophils are a major source of proteases and ROS, being more infiltrated during ischemic brain injury associated with a massive blood influx into the brain and BBB-increased permeability. An elevation in the adhesion of isolated neutrophils to endothelial cells was observed in hyperglycemic diabetic patients compared to normoglycemic subjects [8]. In a mouse model of stroke, we previously found that acute hyperglycemia led to an increase in the cerebral level of myeloperoxidase, used as an indicator of neutrophil recruitment. *A. borbonica* polyphenols and caffeic acid were able to abrogate this detrimental effect of hyperglycemia [12]. Literature data show that there is a significant correlation between neutrophil number and MMP-9 levels in the infarcted hemisphere of obese and diabetic *db*/*db* mice [61]. Here, our in vitro experiments provide evidence that hyperglycemic condition increased the secretion of MMP-2 by cerebral endothelial cells without modulating that of MMP-9. Lucivero et al. [62] demonstrated the differential regulation of MMP-2 and MMP-9 after a human ischemic stroke where MMP-2 is expressed early while MMP-9 is produced lately and related to more severe stroke. In parallel, our in vitro results indicate positive correlations between ROS levels and MMP-2 activity as well as between MCP-1 release and MMP-2 activity. These findings suggest that oxidative stress and inflammation induced by the hyperglycemic condition were associated with increased MMP-2 production by cerebral endothelial cells. This may participate to BBB basal membrane degradation in our model of obese and hyperglycemic mice subjected to stroke. Interestingly, *A. borbonica* polyphenols as well as caffeic acid and quercetin exerted a protective effect by limiting the production of MMP-2 in cerebral endothelial cells exposed to hyperglycemic condition. In rat stroke models, derivatives of caffeic acid such as dihydrocaffeic acid and chlorogenic acid, detected in *A. borbonica*, were able to reduce brain infarct and BBB damage via inhibition of the expression and activities of MMP-2 and MMP-9 in a dose-dependent manner [36,63]. Here, in in vivo condition, the dose of polyphenols from *A. borbonica* and caffeic acid used did not modulate cerebral MMP-2 and MMP-9 levels (data not shown). Nevertheless, polyphenols exhibited anti-inflammatory properties in obese mice after ischemic stroke, by reducing the elevation of pro-inflammatory markers in the brain including IL-1β, IL-6, MCP-1 and TNF-α. In the study by Taïlé et al. [32], we showed that *A. borbonica* polyphenols and caffeic acid decreased the secretion of these pro-inflammatory mediators induced by hyperglycemic condition in cerebral endothelial cells, by down-regulating NFκB activation and by increasing the production of AMP-activated protein kinase (AMPK). Both signaling pathways regulate the inflammatory status and act as opposite pivotal regulators of endothelial functions [32]. In parallel to the decrease in neuroinflammation mediated by *A. borbonica* polyphenols and caffeic acid in obese mice during stroke, the quantities of IL-6 and TNF-α were also lowered by polyphenols in the visceral adipose tissue of obese mice. This is consistent with published data showing the capacity of *A. borbonica* polyphenols and caffeic acid to reduce the secretion of such pro-inflammatory adipokines by inhibiting NFĸB signaling pathway in murine 3T3-L1 adipocytes [34,64].

Literature studies described that astrocyte constitute the predominant glial cells within the brain with a key role on BBB functions. In neuroinflammatory conditions, reactive astrogliosis is associated with an overproduction of GFAP intermediate filament and astrocyte hypertrophy [52,53]. We found that, in the infarcted hemisphere, GFAP levels tended to be more pronounced in obese mice undergoing stroke. This suggests a possible regulation of the morphology/functionality of astrocytes during stroke under obesity condition. Here, polyphenols did not statistically modify GFAP staining. Ashafaq et al. [37] reported that the polyphenol catechin, particularly abundant in green tea, lowered GFAP levels in ischemic rat brain, and correlated this anti-inflammatory action with an improvement of the antioxidant status and a reduction of cerebral infarct size and neurological deficit. The neuroprotective effects of other polyphenols such as kaempferol, detected in *A. borbonica*, on cerebral ischemia-reperfusion damages in vivo were also described. It was found that kaempferol protected against cerebral ischemia-reperfusion-mediated oxidative stress, inflammation and apoptosis. The beneficial effects of kaempferol were related to an increase in the production of the redox-sensitive transcriptional factor Nrf2 that leads to an activation of SOD and GPx antioxidant enzymatic activities and a reduction of TNF-α, IL-1β and IL-6 expression levels in vivo [65]. Ceriello et al. [66] demonstrated an alteration of the activities of Cu/ZnSOD and catalase in models of brain and peripheral endothelial cells exposed to a chronic hyperglycemia during 7–14 days. Our present data indicate that *A. borbonica* polyphenols as well as caffeic acid attenuated oxidative stress aggravated by HFD-induced obesity during stroke. More precisely, polyphenols reduced the overactivation of Nrf2 and SOD enzymatic activity that was specifically detected in the cerebral infarcted hemisphere. Concordantly, our previous data demonstrated a similar result on the cerebral ischemic hemisphere of a mouse model of stroke in acute hyperglycemia [12]. Additionally, in the study by Arcambal et al. [12], we found that polyphenols from *A. borbonica* and caffeic acid inhibited the nuclear translocation of Nrf2 induced by hyperglycemic condition in an in vitro model of cerebral endothelial cells. Our findings are consistent with literature data showing the link between Nrf2 regulation and vascular function improvement in models of Nrf2^−/−^ rats [67] or diabetic rats [68]. In the present study, *A. borbonica* polyphenols and caffeic acid also led to a decrease in the basal SOD activity measured in the cerebral non-infarcted hemisphere that was not changed in obese mice during stroke, showing their global antioxidant potential in the whole brain. This antioxidant protection of polyphenols was also detected in the heart, liver and visceral adipose tissue, suggesting their capacity to improve redox alterations at both the central and peripheral levels in obese mice during stroke. Molecular mechanisms underlying the antioxidant effects of *A. borbonica* polyphenols and caffeic acid may depend on their ability to spare redox mediators such as Nrf2 and SOD. Likewise, *A. borbonica* polyphenols and caffeic acid were reported to improve other ROS-detoxifying enzymes including catalase, GPx and heme oxygenase-1 deregulated by hyperglycemic condition in a mouse model of stroke and cerebral endothelial cells. Oppositely, these polyphenols down-regulated ROS-producing enzymes such as NAPDH oxidases and decreased intracellular ROS levels in cerebral endothelial cells in hyperglycemic condition [12,32]. Such antioxidant properties of *A. borbonica* polyphenols and caffeic acid were also described in in vitro adipocyte models exposed to oxidative stress and pro-inflammatory conditions [34,64]. Furthermore, *A. borbonica* polyphenols including caffeic acid, chlorogenic acid, quercetin and kaempferol were reported to directly scavenge and reduce free radicals [32,34,64]. Thus, altogether, these different molecular mechanisms may contribute to explain the antioxidant benefits of polyphenols in the brain and visceral adipose tissue of obese mice during stroke. It will be relevant to clarify their antioxidant mechanisms of action at the heart and liver levels.

Given that quercetin and caffeic acid were detected in plasma of mice receiving *A. borbonica* polyphenolic extract or caffeic acid, this study suggests their capacity to reach target tissues. Concomitantly, caffeic acid, as well as its methylated metabolite, namely ferulic acid, were previously detected in the infarcted hemisphere, in a mouse model of stroke receiving intraperitoneally *A. borbonica* extract or caffeic acid [12]. Additionally, we reported the uptake of quercetin and caffeic acid by cerebral endothelial cells, with evidence for breast cancer resistance protein (BCRP) efflux transporter involvement [13]. Further studies will help to elucidate the presence of polyphenols in the visceral adipose tissue, heart and liver of obese mice during stroke. Importantly, our present study shows, for the first-time, 10-fold higher circulating levels of hippuric acid in mice receiving caffeic acid or *A. borbonica* polyphenols, suggesting its production from the catabolism of plant polyphenols by the gut bacterial microflora. Indeed, it is well known that hippuric acid originates from the bacterial degradation in the intestinal tract of several polyphenols such as caffeic and chlorogenic acids detected in *A. borbonica* [69,70]. Thus, hippuric acid may contribute to the cerebral and peripheral protective effects depicted during stroke in obese mice exposed to *A. borbonica* polyphenols and caffeic acid. Consistently, our previous data demonstrated that hippuric acid exhibited anti-inflammatory properties in cerebral endothelial cells, by inhibiting the elevation of NFκB activity and IL-6 secretion mediated by hyperglycemic condition [13]. Recently, Brial et al. [71] nicely showed infusion of hippuric acid ameliorated glucose tolerance and insulin secretion in mice exposed to HFD-induced obesity. Moreover, a positive link between hippuric acid urinary levels and improved glucose homeostasis was described in subjects receiving a high-meat diet rich in saturated fats, leading to propose hippuric acid as a novel biomarker and mediator of metabolic health.

Limitations of this study warrant mention. A single dose of 35 mg/kg of polyphenols was tested in mice, in accordance with the average concentration ranging from 10–50 mg/kg broadly used in similar literature studies [36,37,38,39]. Despite the chronic administration of this dose in mice during 12 weeks, it would be relevant to determine a dose-dependent action of *A. borbonica* polyphenols in order to determine the concentration able to reduce the neurological deficit caused by stroke and that was not improved with the dose we selected. A dose-dependent neuroprotective action of caffeic acid has already been reported in a rat model of stroke [36]. Regarding the evaluation of the neurological deficit during stroke, our data indicate non-statistical difference between control and obese mice receiving or not receiving polyphenols. Given that this neurological deficit was calculated according to the method published by Menzies et al. [45], and based on a global score table, it will be interesting to conduct more specific behavioral studies with sensorimotor and cognitive tests as well as the assessment of muscular coordination skill [72]. The neuroprotective action of polyphenols on stroke recovery should also be investigated by exploring their effect on neuron survival and brain reparation. In a rat model of stroke, quercetin was found to reduce brain oedema, and to improve the neurological deficit score evaluating the consciousness, smell, breathing, hearing, vision, motor function and orientation and reflexes. Moreover, quercetin led to an improvement of neuron survival assessed at 24 h after reperfusion and this effect was associated with an amelioration of BBB tight junction ultrastructure [73]. In the study by Ghaddar et al. [74], we showed that *A. borbonica* extract exerted a preventive role against the alteration of brain homeostasis and neurogenesis impairment in a model of diet-induced overweight zebrafish, suggesting its capacity to limit neurological disorders. Taken together, our results provide relevant perspectives for the use of polyphenol-rich extract from *A. borbonica* as an innovative source of bioactive principles in nutritional or pharmaceutical preparations aiming at reducing metabolic and neurological complications aggravated in the context of obesity-related diabetes and stroke.

## 5. Conclusions

This study demonstrates for the first time the protective effects of *A. borbonica* polyphenols and caffeic acid in obese mice exposed to stroke. Polyphenols attenuated the metabolic disorders caused by HFD-induced obesity, including insulin resistance, hyperglycemia, hyperinsulinemia and dyslipidemia. Polyphenols limited cerebral infarct volume, hemorrhagic transformation and BBB dysfunction aggravated by HFD-induced obesity during stroke, via anti-inflammatory and antioxidant effects. These effects may result from the ability of polyphenols to be metabolized and reach the bloodstream. Further studies will be needed to decipher the molecular mechanisms responsible for the protective activities of polyphenols depicted at both the cerebral and peripheral levels, and to assess polyphenol benefits on stroke recovery during obesity and diabetes.

## Figures and Tables

**Table 1:** Effect of HFD and polyphenols on brain and visceral adipose tissue inflammatory markers.

Markers	ND	HFD	HFD + *A.b*	HFD + CA
Total brain				
IL-1β	6.70 ± 0.13	7.56 ± 0.23 *	5.91 ± 0.43 ^$$^	6.04 ± 0.31 ^$$^
MCP-1	2.35 ± 0.70	11.54 ± 2.80 **	3.61 ± 0.19 ^$$^	7.58 ± 0.18
TNF-α	2.40 ± 0.90	3.06 ± 0.90 **	1.83 ± 0.07 ^$$$^	2.02 ± 0.14 ^$$$^
IL-6	1.92 ± 0.16	3.18 ± 0.38 *	1.10 ± 0.29 ^$$$^	1.41 ± 0.28 ^$$^
Infarcted hemisphere				
IL-1β	3.38 ± 0.08	4.13 ± 0.27	3.06 ± 0.38	3.33 ± 0.32
MCP-1	2.02 ± 1.25	10.43 ± 2.34 **	3.23 ± 0.19 ^$^	3.50 ± 0.14 ^$^
TNF-α	1.26 ± 0.22	1.40 ± 0.03	0.94 ± 0.16 ^$^	0.82 ± 0.15 ^$^
IL-6	1.87 ± 0.12	3.01 ± 0.33 *	1.01 ± 0.28 ^$$^	1.34 ± 0.27 ^$$^
Visceral adipose tissue				
IL-1β	0.91 ± 0.09	3.08 ± 0.18 ***	3.01 ± 0.19 ***	2.78 ± 0.20 ***
MCP-1	0.41 ± 0.04	1.26 ± 0.15 ***	1.31 ± 0.10 ***	1.22 ± 0.14 ***
TNF-α	1.10 ± 0.13	3.34 ± 0.30 ***	2.98 ± 0.21 ***	2.26 ± 0.22 *^,$$^
IL-6	0.11 ± 0.01	0.23 ± 0.03 *	0.13 ± 0.02 ^$^	0.13 ± 0.03 ^$^
Leptin	7.57 ± 1.15	32.65 ± 2.55 ***	24.66 ± 2.30 ***^,$^	23.36 ± 2.54 ***^,$^
Adiponectin	31.82 ± 2.63	56.54 ± 5.03 *	92.77 ± 5.16 ***^,$$$^	86.48 ± 4.19 ***^,$$$^

Mice were exposed to a normal diet (ND) or a high-fat diet (HFD) in the presence or not of *A. borbonica* polyphenolic extract (HFD + *A.b*; 35 mg/kg) or caffeic acid (HFD + CA; 35 mg/kg) for 12 weeks before a cerebral ischemia-reperfusion. After a cerebral ischemia of 90 min and 20 h of reperfusion, IL-1β, MCP-1, TNF-α and IL-6 levels (ng) were measured in the brain and visceral adipose tissue by specific ELISA kits. Levels of leptin (ng) and adiponectin (µg) were determined in the visceral adipose tissue using specific ELISA kits. Data were expressed as means ± SEM of n ≥ 5. * *p* < 0.05, ** *p* < 0.01 and *** *p* < 0.005 vs. ND. ^$^ *p* < 0.05, ^$$^ *p* < 0.01 and ^$$$^ *p* < 0.005 vs. HFD.

**Table 2:** Plasma concentrations of polyphenols and related metabolites.

Plasma Level	ND	HFD	HFD + *A.b*	HFD + CA
Caffeic acid	0.357 ± 0.022	0.409 ± 0.026	0.900 ± 0.048 ***^,$$$^	1.227 ± 0.041 ***^,$$$^
Ferulic acid	0.042 ± 0.002	0.046 ± 0.004	0.046 ± 0.002	0.044 ± 0.004
Chlorogenic acid	nd	nd	nd	nd
Quercetin	nd	nd	0.046 ± 0.012 ***^,$$$^	nd
Hippuric acid	0.032 ± 0.009	0.039 ± 0.009	0.357 ± 0.114 ***^,$$$^	0.494 ± 0.110 ***^,$$$^

Mice were exposed to a normal diet (ND) or a high-fat diet (HFD) in the presence or not of *A. borbonica* polyphenolic extract (HFD + *A.b*; 35 mg/kg) or caffeic acid (HFD + CA; 35 mg/kg) for 12 weeks before a cerebral ischemia-reperfusion. After a cerebral ischemia of 90 min and 20 h of reperfusion, plasma levels (µM) of quercetin and caffeic, ferulic, chlorogenic and hippuric acids were measured by mass spectrometry. Data were expressed as means ± SEM of n ≥ 5. *** *p* < 0.005 vs. ND. ^$$$^ *p* < 0.005 vs. HFD. nd: not detected.

## Data Availability

Data is contained within the article.
